# Supplementary figures and images for: SARS-CoV-2 remodels the Golgi apparatus to facilitate viral assembly and secretion
Source: PLoS Pathog. 2025 Jun 20;21(6):e1013295. doi: 10.1371/journal.ppat.1013295 (PMC12208438; doi:10.1371/journal.ppat.1013295)

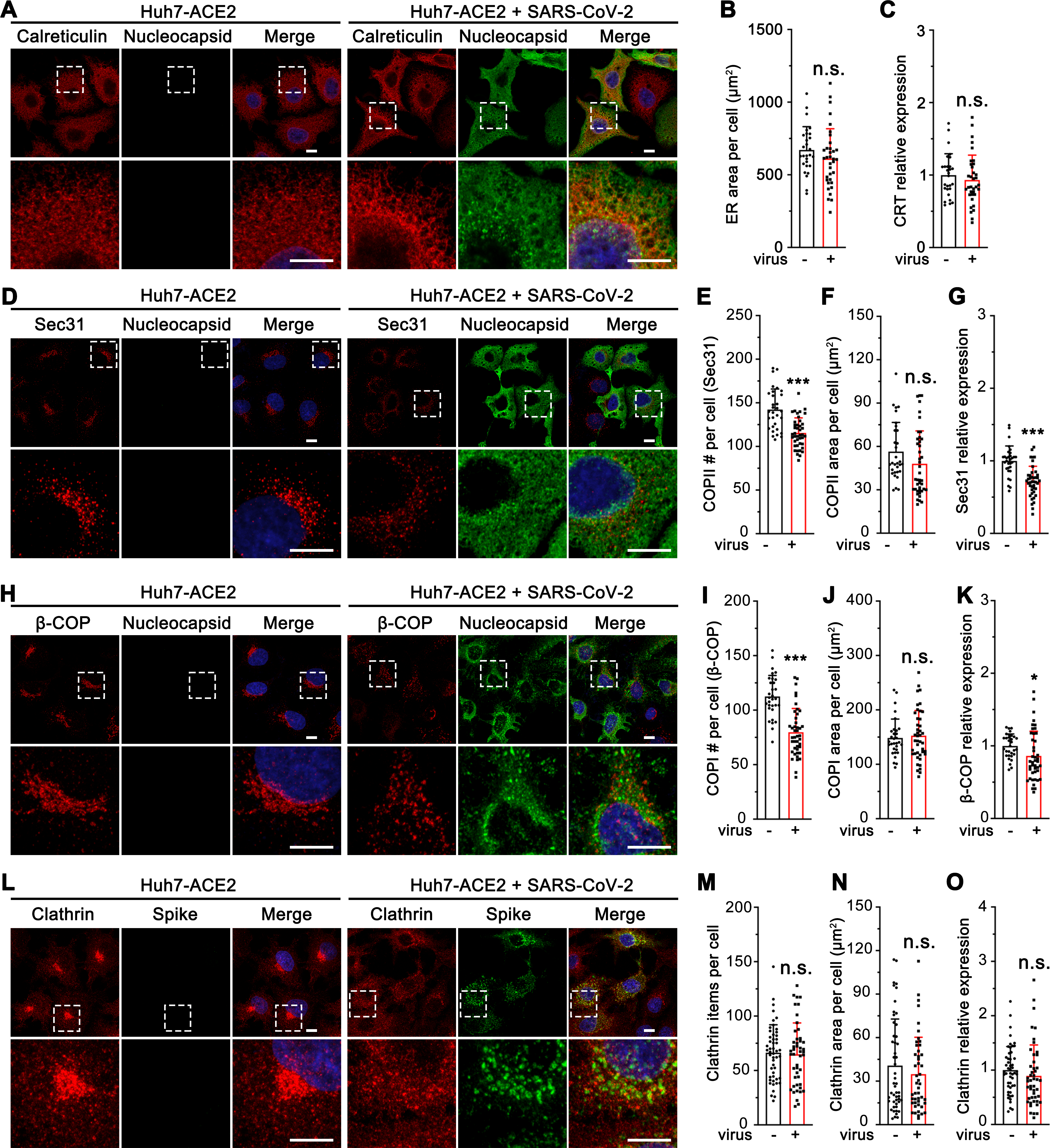

Supplement: S1 Fig — (A) Representative confocal images of Huh7-ACE2 cells incubated with or without SARS-CoV-2 (MOI = 1) for 24 h and stained for calreticulin and nucleocapsid. (B-C) Quantification of calreticulin for the area (B) and relative expression (C) in A. (D) Representative confocal images of Huh7-ACE2 cells incubated with or without SARS-CoV-2 (MOI = 1) for 24 h and stained for Sec31 and nucleocapsid. (E-G) Quantification of Sec31 item number (E), area (F), and relative expression (G) in D. (H) Representative confocal images of Huh7-ACE2 cells incubated with or without SARS-CoV-2 (MOI = 1) for 24 h and stained for β-COP and nucleocapsid. (I-K) Quantification of β-COP in H. (L) Representative confocal images of Huh7-ACE2 cells incubated with or without SARS-CoV-2 (MOI = 1) for 24 h and stained for clathrin and spike. (M-O) Quantification of clathrin in L. Boxed areas in the upper panels are enlarged and shown underneath. Scale bars in all panels, 10 μm. All quantitation data are shown as mean ± SD from three independent experiments. Statistical analyses were performed using two-tailed Student’s t-test. *, p < 0.05; ***, p < 0.001; n.s., not significant. (TIF) [file ppat.1013295.s001.tif]

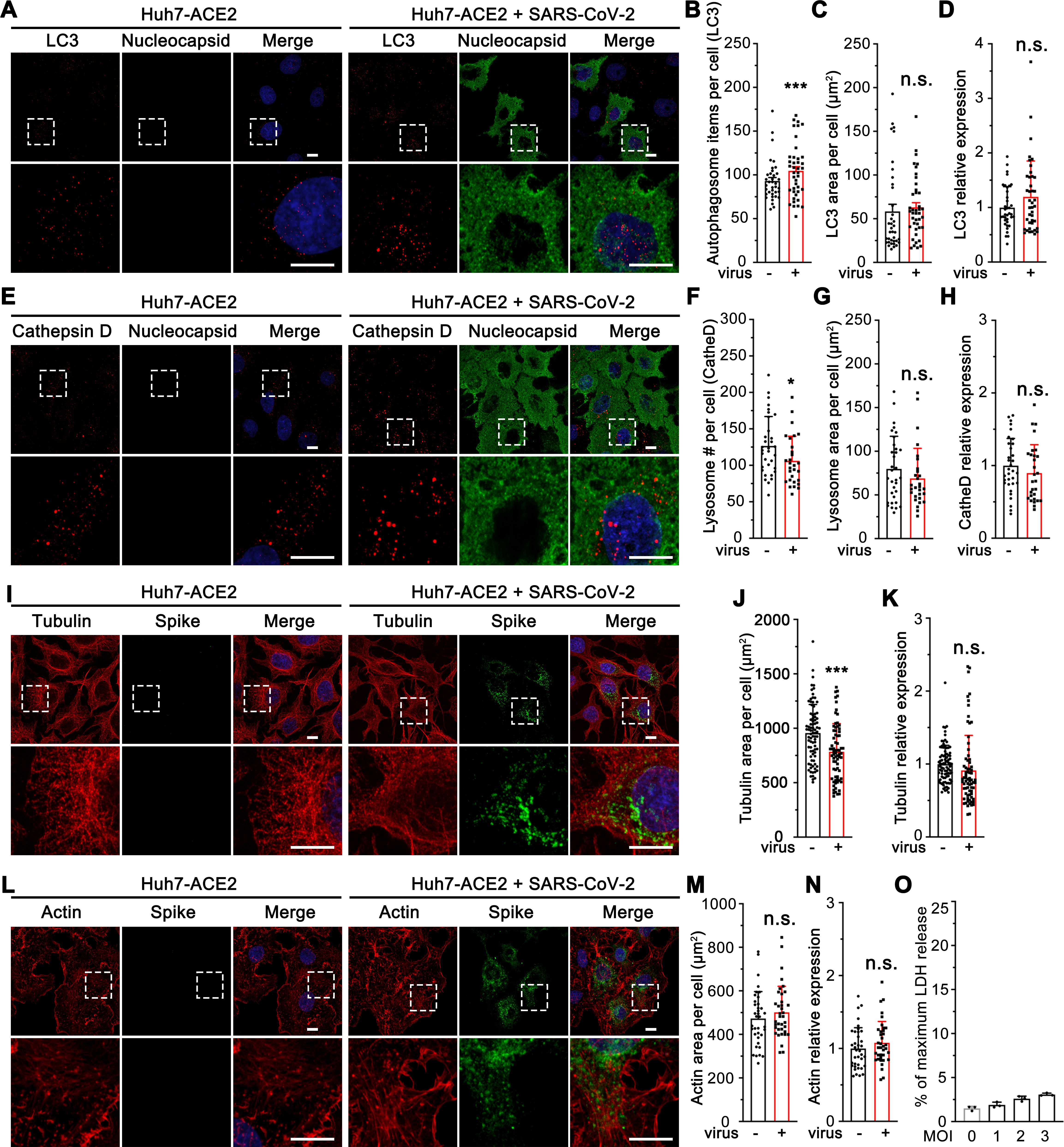

Supplement: S2 Fig — (A) Representative confocal images of Huh7-ACE2 cells incubated with or without SARS-CoV-2 (MOI = 1) for 24 h and stained for LC3 and nucleocapsid. (B-D) Quantification of LC3 item number (B), area (C), and relative expression (D) in A. (E) Representative confocal images of Huh7-ACE2 cells incubated with or without SARS-CoV-2 (MOI = 1) for 24 h and stained for cathepsin D and nucleocapsid. (F-H) Quantification of cathepsin D in E. (I) Representative confocal images of Huh7-ACE2 cells incubated with or without SARS-CoV-2 (MOI = 1) for 24 h and stained for α-Tubulin and spike. (J-K) Quantification of tubulin in I. (L) Representative confocal images of Huh7-ACE2 cells incubated with or without SARS-CoV-2 (MOI = 1) for 24 h and stained for actin (with phalloidin) and spike. (M-N) Quantification of actin in L. Boxed areas in the upper panels are enlarged and shown underneath. Scale bars in all panels, 10 μm. (O) Representative LDH assay of Huh7-ACE2 cells infected with SARS-CoV-2 at different MOIs for 24 h with 4 technical replicates from two independent experiments. All quantitation data are shown as mean ± SD from three independent experiments. Statistical analyses were performed using two-tailed Student’s t-test. *, p < 0.05; ***, p < 0.001; n.s., not significant. (TIF) [file ppat.1013295.s002.tif]

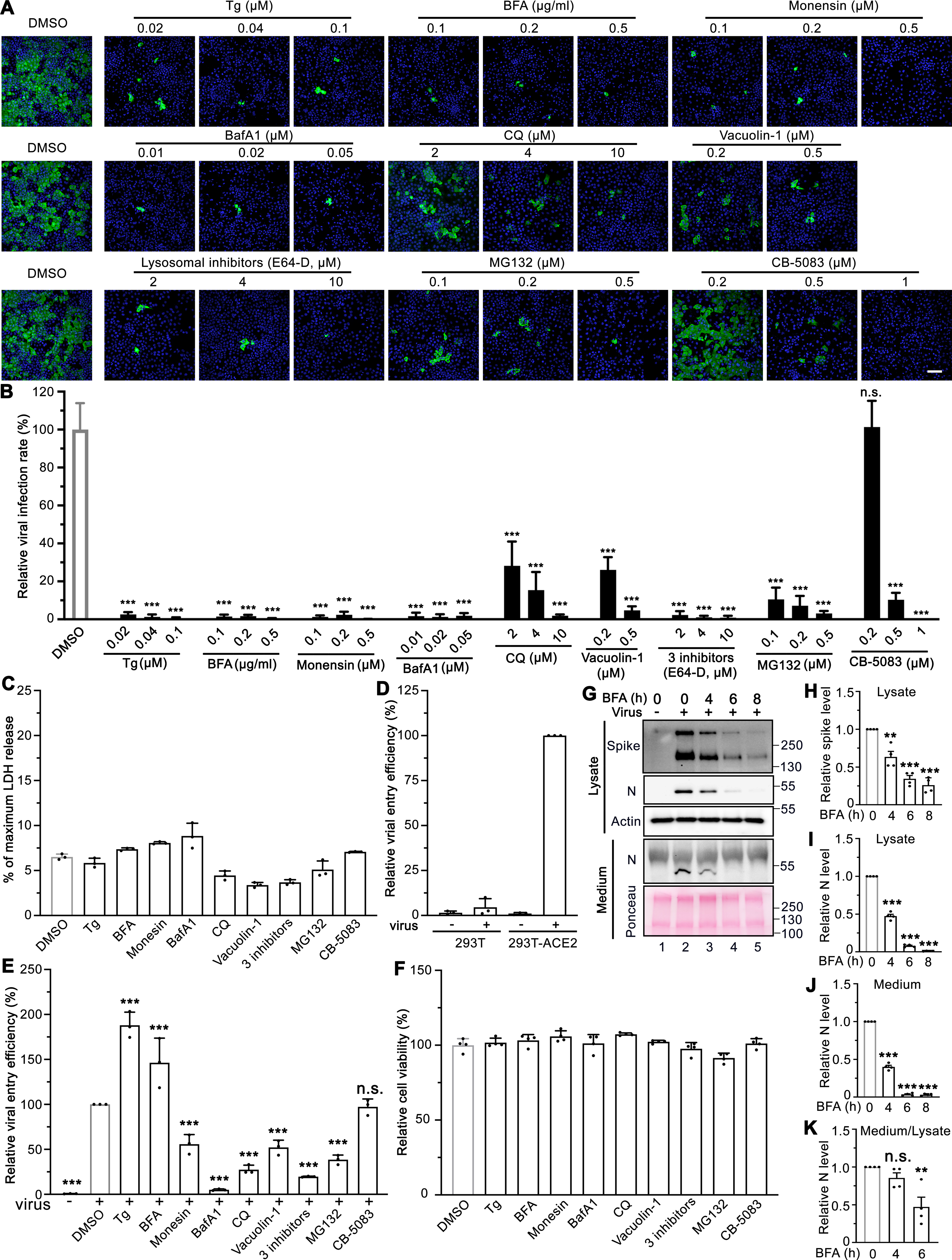

Supplement: S3 Fig — (A) Representative confocal images of Huh7-ACE2 cells infected with SARS-CoV-2 for 24 h in the presence of indicated molecules and stained for nucleocapsid. Scale bar, 100 μm. Quantification of the viral infection percentage is shown in Fig 2C. (B) Quantification of the viral infection percentage in the presence of indicated chemicals with different concentrations of A, with the control normalized to 100%. Data are shown as mean ± SD from 10 representative images. (C) Representative LDH assay of Huh7-ACE2 cells treated with the indicated molecules at the same concentrations as in Fig 2A and infected with SARS-CoV-2 (MOI = 1) for 24 h with 3 technical replicates from two independent experiments. (D) Validation of cell entry assay of 293T and 293T-ACE2 cells by infection of SARS-CoV-2 Spike pseudotyped lentiviruses. (E) Cell entry assay of 293T-ACE2 cells by SARS-CoV-2 spike pseudotyped lentivirus for 24 h in the presence of indicated molecules at the same concentrations as in Fig 2A. Data are shown as mean ± SD from three independent experiments. Statistical analyses are performed using One-way ANOVA. ***, p < 0.001, n.s., not significant. (F) Cell viability assay of 293T-ACE2 cells treated with indicated molecules for 24 h. (G) Immunoblots of cell lysates and media of Huh7-ACE2 cells incubated with or without SARS-CoV-2 (MOI = 3) for 12 h and treated with 5 µg/ml BFA for the indicated time points. (H-K) Quantification of intracellular spike protein (H), intracellular N protein (I), extracellular N protein (J), and medium/lysate ratio of N protein (K) in G. Data are presented as mean ± SD from 4 replicates of two independent experiments. (TIF) [file ppat.1013295.s003.tif]

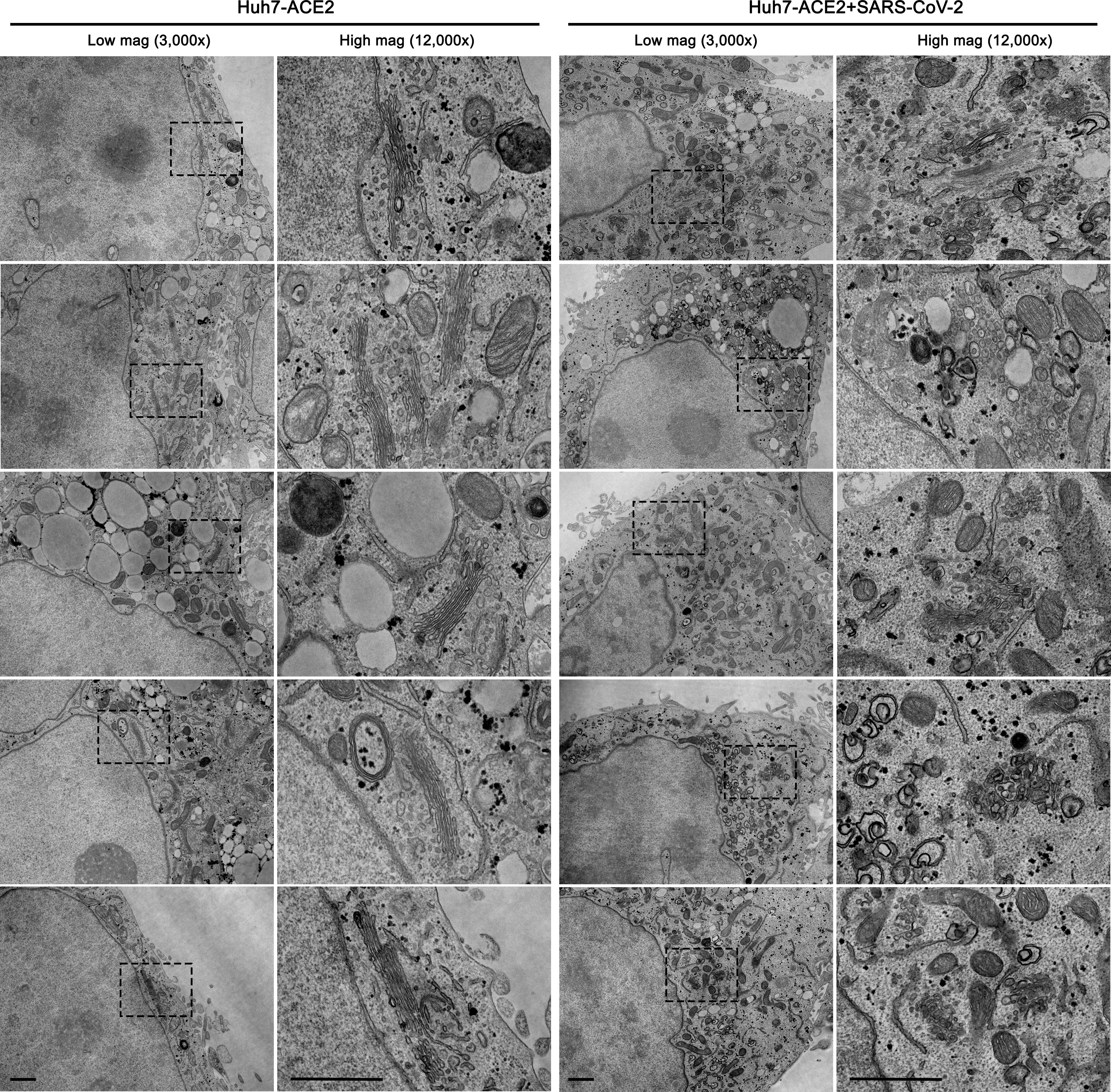

Supplement: S4 Fig — A gallery of EM images of Huh7-ACE2 cells incubated with or without SARS-CoV-2 (MOI = 1) for 24 h under two different magnifications. Boxed areas on the left images are enlarged and shown on the right. Scale bars, 500 nm. (TIF) [file ppat.1013295.s004.tif]

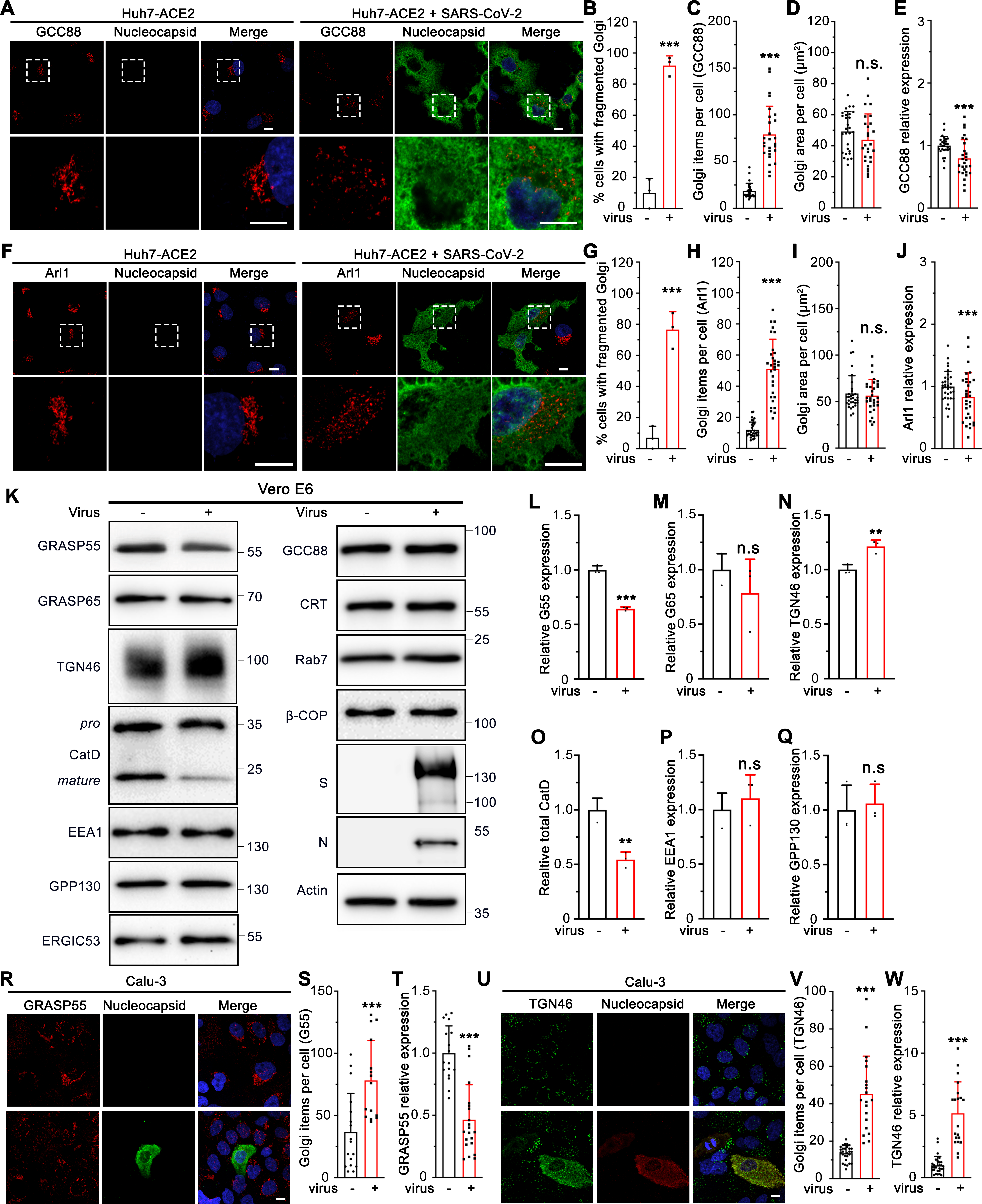

Supplement: S5 Fig — (A) Representative confocal images of Huh7-ACE2 cells incubated with or without SARS-CoV-2 (MOI = 1) for 24 h and stained for a trans-Golgi marker GCC88 and nucleocapsid. (B-E) Quantification of A for the percentage of cells with fragmented Golgi (B), GCC88 item number (C), area (D), and relative expression level (E). (F) Representative confocal images of Huh7-ACE2 cells incubated with or without SARS-CoV-2 (MOI = 1) for 24 h and stained for a trans-Golgi marker Arl1 and nucleocapsid. Boxed areas in the upper panels of A and F are enlarged and shown underneath. Scale bars, 10 μm. (G-J) Quantification of Arl1 in F. (K) Immunoblots of indicated proteins in Vero E6 cells incubated with or without SARS-CoV-2 (MOI = 1) for 24 h. (L-Q) Quantification of K for the relative level of GRASP55 (L), GRASP65 (M), TGN46 (N), cathepsin D (CatD, O), EEA1 (P), and GPP130 (Q). (R) Representative confocal images of Calu-3 cells incubated with or without SARS-CoV-2 (MOI = 1) for 24 h and stained for a medial/trans-Golgi marker GRASP55 and nucleocapsid. (S-T) Quantification of R for the GRASP55 item number (S) and relative expression level (T). (U) Representative confocal images of Calu-3 cells incubated with or without SARS-CoV-2 (MOI = 1) for 24 h and stained for a TGN marker TGN46 and nucleocapsid. (V-W) Quantification of R for the TGN46 item number per cell (V) and relative expression level (W). Quantitation data are shown as mean ± SD from at least three independent experiments. Statistical analyses were performed using two-tailed Student’s t-test. **, p < 0.01; ***, p < 0.001; n.s., not significant. (TIF) [file ppat.1013295.s005.tif]

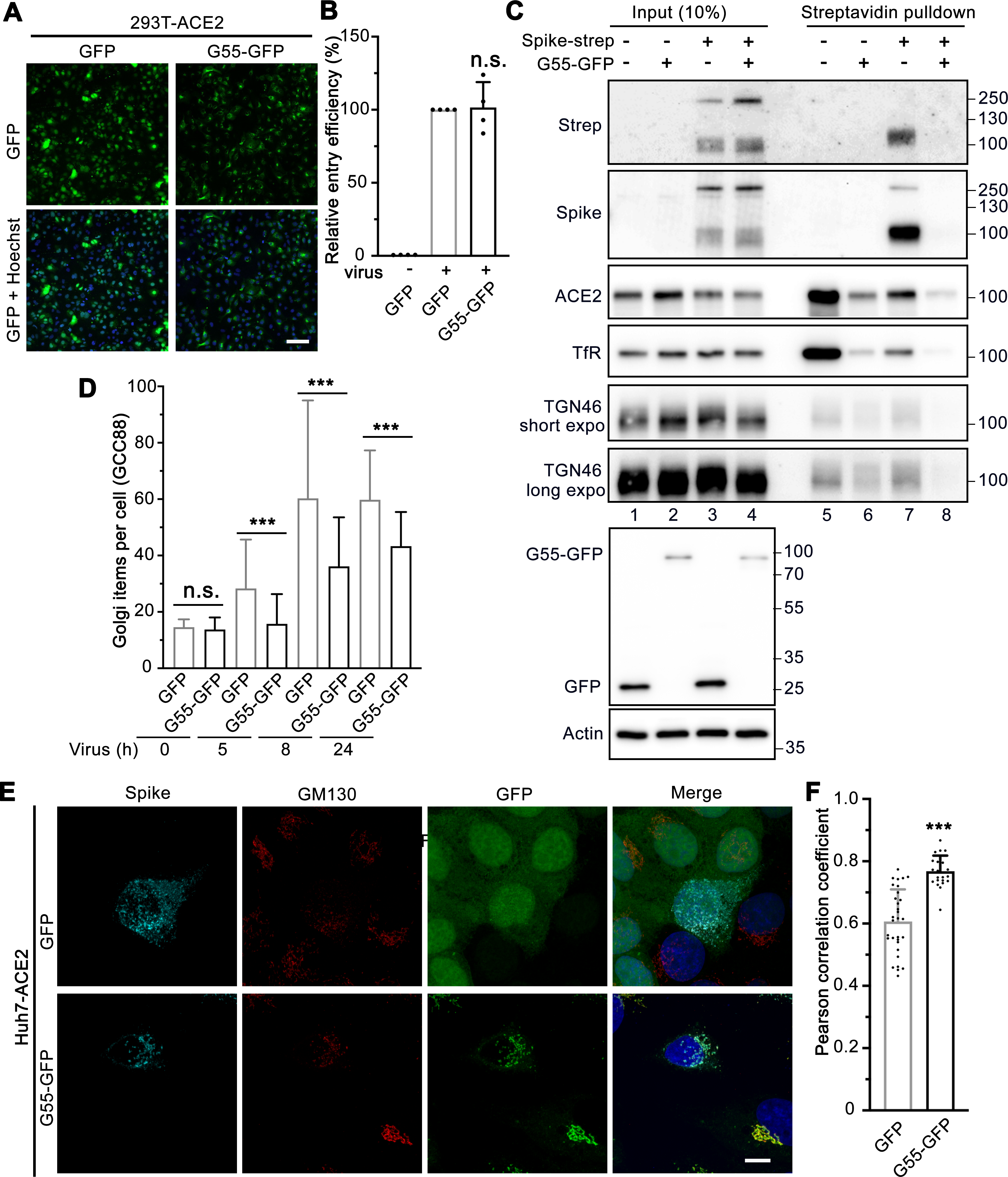

Supplement: S6 Fig — (A) Representative confocal images of stable 293T-ACE2 cells expressing GFP or GRASP55-GFP. Scale bar, 100 μm. (B) Cell entry assay of 293T-ACE2 cells stably expressing either GFP or GRASP55-GFP by SARS-CoV-2 spike pseudotyped lentivirus. (C) GRASP55 expression reduced spike at the cell surface. Huh7-ACE2 cells were transfected with GFP or GRASP55-GFP for 24 h, and then co-transfected with GFP or GRASP55-GFP together with spike-strep for 24 h. Cell surface proteins were biotinylated, pulled down by streptavidin beads, and blotted for indicated proteins. (D) Quantification of the Golgi item number of GCC88 of Huh7-ACE2 cells stably expressing GFP or GRASP55 infected by SARS-CoV-2 for 5, 8, and 24 h. Data are shown as mean ± SD from more than 24 random images from two independent experiments. (E) Representative confocal images of Huh7-ACE2 cells stably expressing GFP or GRASP55-GFP infected by SARS-CoV-2 for 24 h. Scale bar, 10 μm. (F) Pearson correlation coefficient between spike and GM130 in E. Statistical analyses were performed using two-tailed Student’s t-test. ***, p < 0.001; n.s., not significant. (TIF) [file ppat.1013295.s006.tif]

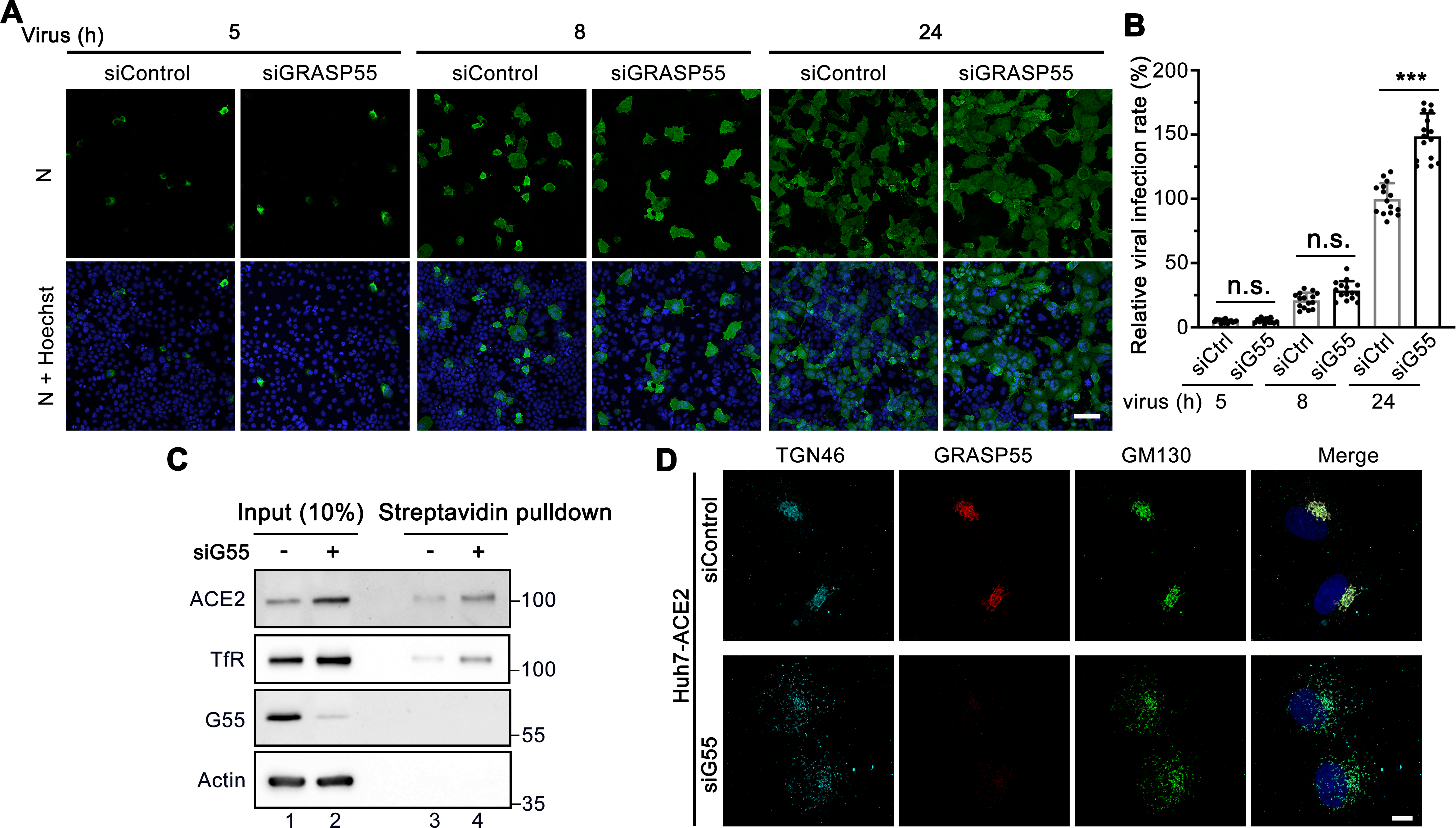

Supplement: S7 Fig — (A) Representative confocal images of Huh7-ACE2 cells transfected with siControl or siGRASP55 oligos for 48 h followed by infection with SARS-CoV-2 for 5, 8, and 24 h. Scale bar, 100 μm. (B) Quantification of the viral infection percentage in B. Data are shown as mean ± SD from 30 random images from two independent experiments. Statistical analyses were performed using One-way ANOVA. ***, p < 0.001; n.s., not significant. (C) GRASP55 depletion increased ACE2 level at the cell surface. Huh7-ACE2 cells were transfected with siControl or siGRASP55 RNAi oligoes for 72 h. Cell surface proteins were biotinylated, pulled down by streptavidin beads, and blotted for indicated proteins. (D) Representative confocal images of Huh7-ACE2 cells transfected with siControl and siGRASP55 RNAi oligoes. Scale bar, 10 μm. (TIF) [file ppat.1013295.s007.tif]

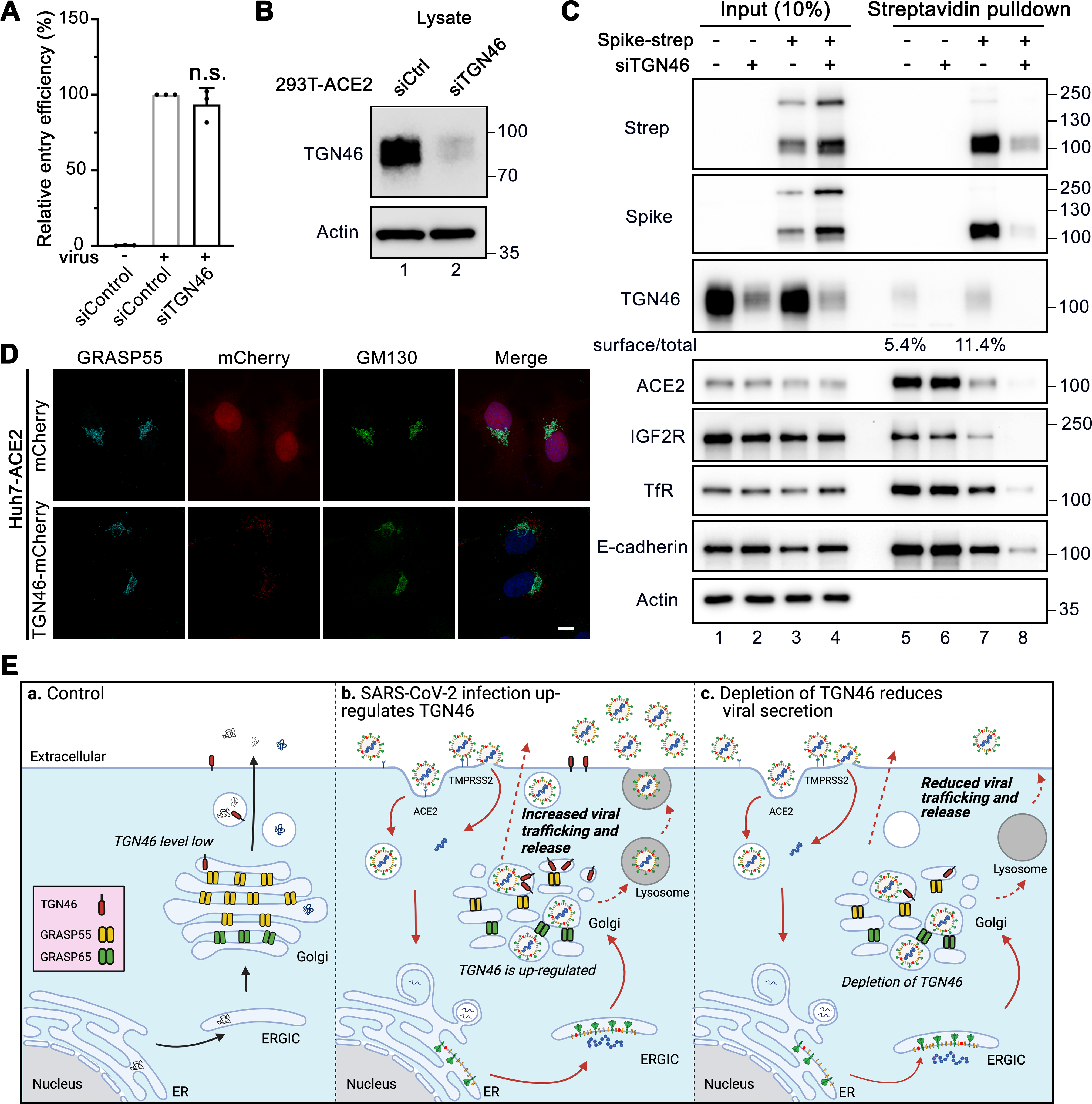

Supplement: S8 Fig — (A) Cell entry assay of 293T-ACE2 cells transfected with siControl or siTGN46 oligos for 48 h followed by infection with SARS-CoV-2 spike pseudotyped lentivirus for 24 h. Statistical analyses were performed using two-tailed Student’s t-test. n.s., not significant. (B) Immunoblots of cell lysates of 293T-ACE2 cells transfected with siControl or siTGN46 oligos for 48 h followed by infection with SARS-CoV-2 spike pseudotyped lentivirus for 24 h. (C) TGN46 depletion reduces spike protein at the cell surface. Huh7-ACE2 cells were transfected with siControl or siTGN46 oligos for 48 h followed by transfection with spike-strep for 24 h. Cell surface proteins were biotinylated, pulled down by streptavidin beads, and blotted for indicated proteins. Note that TGN46 level is enhanced at the cell surface by spike protein expression. (D) Representative confocal images of Huh7-ACE2 cells stably expressing mCherry and TGN46-mCherry. Scale bar, 10 μm. (E) Proposed working model for a novel role of TGN46 in SARS-CoV-2 infection. In brief, under normal conditions (a) TGN46 is expressed at a relatively low level and recycles between the Golgi and plasma membrane. After SARS-CoV-2 infection (b), TGN46 is up-regulated, which accelerates viral trafficking. When TGN46 is depleted (c), the trafficking speed of all variants of SARS-CoV-2 is reduced. Thus, TGN46 may serve as a carrier for viral trafficking and release. Created with BioRender. (TIF) [file ppat.1013295.s008.tif]
