# Supplementary material for: SARS-CoV-2 remodels the Golgi apparatus to facilitate viral assembly and secretion
Source: PLoS Pathog. 2025 Jun 20;21(6):e1013295. doi: 10.1371/journal.ppat.1013295 (PMC12208438; doi:10.1371/journal.ppat.1013295)
Supplement: Attachment — Submitted filename: 146555_1_rebuttal_3318185_ssq2x5.pdf [file ppat.1013295.s013.pdf]

Full Revision

Review COMMONS

Manuscript number: RC-2024-02482
Corresponding author(s): Jianchao Zhang; Andrew Tai; Yanzhuang Wang

Point-by-point description of the revisions

This section is mandatory. Please insert a point-by-point reply describing the revisions that were already carried out and included in the transferred manuscript.

Reviewer #1

Evidence, reproducibility and clarity

In this manuscript, the authors highlight the importance of the Golgi apparatus during SARS-CoV-2 infection. Specifically, using different compounds able to alter Golgi structure and function, the authors show a strong reduction in SARS-CoV-2 infection rate. In particular it is interesting to observe that treatments of 24 hrs with BFA strongly impair viral infection, highlithing the importance of Golgi function for this virus. Albeit the time of treatment is different. this observation is in contrast with previous studies on related coronaviruses (Ghosh et al., 2020) that did not observe any effect upon treatment with BFA. This might imply that SARS-CoV-2 relies more on conventional trafficking pathways respect to other coronaviruses which, under certain conditions, favour different trafficking routes.

We thank the reviewer for the positive comments. Indeed, our results with BFA treatment for 24 hours are inconsistent with previous studies based on the prototype coronavirus MHV (Ghosh et al., 2020). To validate this observation, we have now performed new experiments with BFA treatment for 4, 6, and 8 hours, matching the time points used in the previous study (Ghosh *et al*, 2020). Our new results show that BFA treatment at these early time points significantly inhibits SARS-CoV-2 assembly and secretion, as measured by immunoblotting and TCID50 assays, without reducing intracellular viral RNA levels, which serve as a marker of genome replication. This implies that Golgi function and an intact ER-to-Golgi trafficking route are required for SARS-CoV-2 assembly and secretion. These new results are now presented as new Fig. 2C-H.

The authors additionally observed that viral infection increases TGN46 levels while decreasing GRASP55 levels. To dissect the role of TGN46 and GRASPR55, the authors performed several infection studies in cells in which the levels of the two proteins were modulated either by overexpression (GRASP55) and/or siRNA-mediated knock-down (GRASP55 and TGN46). Those approaches suggest that GRASPR55 overexpression, a protein essential for Golgi stack formation, decelerates viral trafficking and inhibits viral assembly while its depletion reverses the effects. On the other hand, TGN46 knock-down impairs viral trafficking but not assembly. Overall the study clearly shows the importance of the Golgi during SARS-CoV-2 and also shows that modulation of those two factors affect viral infection.

Full Revision

We appreciate the reviewer's accurate summary of our work and positive comments.

However the claims that specifically the trafficking (TGN46) and trafficking and assembly (GRASP55) are not fully substantiated.
Regarding GRASP55, the authors state that viral infection decreases GRASPR55 levels and this results in Golgi fragmentation. However GRASPR55 levels decrease is shown at 24 hrs post infection while Golgi fragmentation occurs as early as 5 hrs. Thus there might be no direct casual effect between the two effects.

We agree with the reviewer that GRASP55 downregulation is unlikely to be the only reason for Golgi fragmentation in the infected cells. In our results, 5- or 8-hour post infection caused only mild Golgi fragmentation (Fig. S6D), while 24 hours post infection led to severe Golgi fragmentation. On the other hand, GRASP55 is likely to play a relevant role as SARS-CoV-2 induced Golgi fragmentation can be partially rescued by exogenous GRASP55 expression (Fig S6C). We have modified the text in lines 303-305 accordingly to acknowledge the possibility that other factors also contribute to Golgi fragmentation in infected cells.

Additionally, the authors show that overexpression of GRASP55 rescue Golgi fragmentation, as observed by imaging, however is not clear if only infected cells where quantified and if they had the same level of infection.

Yes, only infected cells with either GFP or GRASP55-GFP expression were quantified. The viral infection rate was significantly lower in GRASP55-GFP expressing cells compared to GFP expressing cells (Fig 5A-B).

The authors exclude and effect on entry based on experiment on Spike expressing pseudovirus in 293-ACE2, however they also clearly observe reduction of ACE2 on the membrane of GRASPR55 expressing cells (Fig S6B). Thus how can they explain this discrepancy and how ca defect in entry can be fully marked out in these cell lines?

We thank the reviewer for pointing this out. This discrepancy is likely due to the different systems used in the two experiments.
In the pseudovirus entry assay, ACE2 was exogenously expressed in 293T cells and GRASP55 expression did not show any effect on the viral entry efficiency. In contrast, Huh7-ACE2 cells were selected for a high surface expression of ACE2. While GRASP55 expression reduces surface ACE2 levels as shown in our cell surface biotinylation assay, we believe that the surface ACE2 levels in GRASP55-expressing cells remain sufficient to support viral entry. To further investigate whether GRASP55 expression affects viral entry using authentic SARS-CoV-2, we performed RT-qPCR analysis of intracellular RNA level of the spike, N, and RdRp in both GFP and GRASP55-GFP expressing cells 4 hours post infection (new Fig 5D). Our results show that GRASP55 expression does not affect SARS-CoV-2 entry efficiency, even though it reduces ACE2 surface expression levels.

It is not clear to which process the authors refer to when they write about "viral trafficking". Is it virion trafficking or viral proteins trafficking? The two process are linked but are not the same. This oversemplification can be misleading. For instance the authors show that overexpression of GRASP55 decreases Spike protein on the plasma membrane and its depletion increases S protein incorporation into psudoviruses. However it was shown that in infected cells S protein is mainly retained at the ERGIC by M and E (Boson et al., 2021) where viral assembly occurs. Thus an increase in S trafficking on the PM does not correlate with an increase in virion trafficking,

We agree with the reviewer that our use of the term “viral trafficking” is imprecise and we have changed this throughout the manuscript to be more specific. S trafficking to the PM may not necessarily be equal to an increase in virion trafficking and thus have rephrased these terms in our writing accordingly.

We acknowledge that our cell surface biotinylation assay results only demonstrate that GRASP55 overexpression slows down spike protein trafficking to the PM. We have accordingly also examined viral protein and infectious particle secretion into the culture medium as a more direct readout of virion trafficking (new Fig 2E, 2H, 6K, and 7P).

Finally, we have removed all of the data describing spike incorporation into pseudoviruses as we acknowledge that plasma membrane assembly of lentiviruses is not a good model for SARS-CoV-2 assembly.

…and ultimately, the data provided do not fully support the authors claim on a modulation of "virion trafficking" in response to GRASP or TGN46 changes, since no experiments clearly show a change in virions secretion.

In response to the above comment, we provide the following clarification: Our Western blotting, TCID50 assay, and plaque assay results collectively demonstrate that SARS-CoV-2 virion secretion is reduced in GRASP55 expressing cells (new Fig 5E-M) and in TGN46-depleted cells (new Fig 7F-H, 7L-N). Conversely, viral assembly and secretion appear to be increased in GRASP55-depleted cells (new Fig 6A, 6E-I) at 24 hpi. Furthermore, within a single viral secretion cycle (10 hpi), GRASP55 depletion increased viral secretion (new Fig 6K), while TGN46 depletion reduced viral secretion (new Fig 7P). These findings strongly support the conclusion that GRASP55 and TGN46 modulate viral secretion.

Importantly, the authors do not rule out potential effects of their perturbations on genome replication. The only experiment that they perform in this direction is presented in Fig. S7B, where the authors show similar percentage of infected cells at early stage upon silecing of GRASPR55. The experiment suggests that productive entry is similar in these conditions, but quantification of intracellular viral genome could exclude a change in viral replication. If no changes in viral replication are observed, the authors could verify an increase in particles

secretion by collecting supernatants from the early time points and performing plaque assays and quantification of viral genomes by qRT-PCR, to prove that modulation of GRASPR55 indeed promote SARS-CoV-2 trafficking.

We thank the reviewer for the excellent suggestions. In response, we performed RT-qPCR analysis in GRASP55-expressing and TGN46-depleted cells at 4 hpi to compare the viral genome replication process. Additionally, we performed western blotting analysis and released viral titer assay of the culture media from both GRASP55-depleted and TGN46-depleted cells at 10 hpi to investigate virion release. Our new results show that GRASP55 depletion increases viral secretion (new Fig. 6K), while TGN46 depletion reduces viral secretion (new Fig. 7P). Furthermore, GRASP55 expression and TGN46 depletion do not perturb viral genome replication (new Fig. 5D and new Fig. 7R).

Finally, whenever reduction of viral infection is observed upon cell partubation, a robust analysis of cell viability should be presented to exclude pleiotropic effects. Expecially in presence of multiple pertubation that might affect cell metabolism. The authors should carefully control cell viability and growth in response to depletion of TGN46 and GRASP55.

We thank the reviewer for the excellent suggestions, which were also pointed out by reviewer #3. To address this, we performed the LDH cytotoxicity assay under SARS-CoV-2 infection conditions with TGN46 depletion and GRASP55 depletion/expression (new Fig. 5C, 6L, 7Q). Our new results show that no significant cell death was induced by TGN46 depletion, GRASP55 depletion/expression, or other perturbations.

Minor:
show data on viability of the drug and add the relative section in Material and Methods.

We performed LDH assays of SARS-CoV-2 infected Huh7-ACE2 cells treated with 9 small molecules, and LDH release levels were similar across all treatments (new Fig. S3C). Additionally, a CellTiter Glo viability assay of 293T-ACE2 cells did not show any significant effect of cell viability with small molecule treatment (new Fig S3F). Detailed descriptions of these assays have been included in the Material and Methods section.

Figure 3A: should read spike and not nucleocapsid
eported for SARS-CoV-2

Fig. 3A labeling is correct – cells were labeled with antibodies for GRASP65 (rabbit) and for nucleocapsid (mouse).

Lack of inhibition with camostat correlates with lack of TMPRSS2 in the Huh7. The sentence seems to be too general while in this case the effect is clearly cell specific. Similarly, the importance of the lysosome in viral entry is restricted to cells lacking TMPRSS2 and cannot be generalized since CQ, for example, does not work in Calu-3 cells that express TMPRSS2 cells.

We agree with the reviewer and have added one sentence: The relative smaller effect of camostat mesylate observed here, compared to previous studies (Hoffmann et al, 2021), might be due to the use of different cell lines across studies in lines 182-184. We also discussed the discrepancy of CQ treatment between our Huh7-ACE2 cells and Calu-3 cells (Hoffmann et al, 2020) in lines 466-473.

Typo: Fig S3B - Y axis should reat viral not vrial

Thank you – we have corrected this.

S3C: concentrations of the compound used in the assay should be reported. Was a viability assay performed also in the 293T-ACE2 cell line?

We thank the reviewer for the suggestion. We have added the concentration information to the legend in Fig. S3E "Cell entry assay of 293T or 293T-ACE2 cells by SARS-CoV-2 Spike pseudotyped lentivirus for 24h in the presence of indicated molecules at the same concentrations as in Fig. 2A." Additionally, we performed a CellTiter Glo assay to assess the viability of 293T-ACE2 cells treated with the 9 molecules. The results demonstrate that treatment with these 9 molecules does not alter cell viability (Fig. S3F).

Significance

Overall, the major strenght of the manuscript is that it has clarified the importance of the Golgi during SARS-CoV-2 infection. The drugs screening demonstrate that for SARS-CoV-2 the conventional secretion seems to have major role respect to other secretory routes observed for other coronaviruses. Also it is clear that the two factors identified by the authors have a role in viral infection, however the major limitation is that the authors failed to clearly highlight which step/s of the viral life cycle are modulated upon GRASP55 and TGN46 perturbatio. Expecially the claims on "trafficking" is not fully substantiated, since the only experiment in this direction is the transport of Spike protein on the plasma membrane upon GRASPR55 overexpression. It is risky to conclude that the trafficking of a single protein reflect the intracellular trafficking of the virions.

Several of the finding presented in the first part of the manuscript have been already previously reported (for example the fragmentation of the Golgi upon SARS-CoV-2 infection), however the role of GRASP55 and TGN46 in SARS-CoV-2 infection has been reported here for the first time. This manuscript can be of interest for a broad audience considering the topic (cell biology, host-pathogen interactions and molecular virology)

My expertise reside in the field of molecular virology, expecially in the contest of the mechanisms of viral replication and host-pathogen interactions.

Full Revision

We thank the reviewer for the overall positive comments and excellent suggestions. We hope that our new results have convincingly demonstrated that viral trafficking is regulated by GRASP55 and TGN46.
Reviewer #2 (Evidence, reproducibility and clarity (Required)):

Summary:
In this study, Zhang and colleagues address the impact on SARS-CoV-2 infection on the morphology of the Golgi apparatus and convincingly demonstrate a fragmentation of this organelle in infected cells. Conversely, they show that the modulation of TGN46 or GRASP55 expressions, two components of this organelle impact SARS_CoV-2 replication. By monitoring the relative levels of viral Spike and nucleocapsid in the cell supernatants, they conclude that GRASP55 regulates particle assembly and trafficking while TGN46 controls only secretion. The study was generally well performed, and the quality of the microscopy and western blot data is good. It was appreciated that all the phenotypes were robustly quantified. I believe that this study is potentially interesting and relevant for the SARS-CoV-2 community since providing an extensive characterization of the interplay between SARS-CoV-2 and the Golgi apparatus.

We thank the reviewer for the positive comments.

However, as described below, I have some concerns regarding the interpretations of some of the key conclusions. Moreover, the fact that it was already described by several groups that Golgi is a key machinery used for SARS-CoV-2 virion assembly (ERGIC) and secretion dampens my enthusiasm about the study, especially without clear molecular mechanisms about the interplay between SARS-CoV-2 proteins and TNG46/GRASP55.

We rephrased some sentences following the reviewer's suggestions. Although it was believed that SARS-CoV-2 is assembled at the ERGIC, there has been significant controversy surrounding the virion secretion pathway. Our results strongly support that SARS-CoV-2 virions traffic through the Golgi apparatus and that an intact ER-to-Golgi trafficking pathway is essential for SARS-CoV-2 assembly and secretion. Manipulation of two Golgi-resident proteins, GRASP55 and TGN46, significantly regulates SARS-CoV-2 secretion. Interestingly, GRASP55 regulates both assembly and secretion of SARS-CoV-2, while TGN46 exclusively modulates viral secretion. This is consistent with their subcellular localization, as GRASP55 is localized to the medial/trans Golgi, whereas TGN46 is localized to the TGN. We hope that our new experimental results (Figs. 2C-H, 5C-D, 6J-L, and 7O-R) have addressed all concerns from the reviewer. Identification of downstream protein targets involved in TGN46/GRASP55-mediated modulation of SARS-CoV-2 trafficking will be the focus of our future studies.

Major comments:
-All the assays have been performed in liver-derived Huh7 cells (overexpressing SARS-CoV-2 receptor) ACE2 (for infection) or kidney 293 cells (for pseudotyped HIV entry assays). However, no conclusion was validated in lung-derived cells (like A549-ACE2, Calu-3 or primary cells), which would be important since the respiratory tract is the main target of SARS-CoV-2

In our study, Huh7-ACE2 cells are sorted for the high expression of endogenous ACE2 protein, and we did not overexpress ACE2 protein. Also, the liver has been reported to be a site of SARS-CoV-2 infection in humans (Barnes, 2022). We did use A549 and Calu-3 cells in pilot experiments; A549 cells displayed infection rates that were too low for our purposes, and Calu-3 cells showed both low infection rates and relatively disorganized Golgi in the absence of viral infection. We were able to add new IF results from Calu-3 cells. Consistent with our findings in Huh7-ACE2 cells, SARS-CoV-2 infection disrupts Golgi structure and alters protein levels of TGN46 and GRASP55 in Calu3 cells (new Fig. S5R-W). We also confirmed GRASP55 downregulation and TGN46 upregulation in VeroE6 cells (Fig S5K-N).

-Fig2: The impact of the drugs on replication was assessed by measuring the % of infected cells. At 24 hpi, I am unsure about what this value is supposed to measure (the whole life cyle, intracellular replication or spread?), especially since it is not indicated when the drugs were added to the cells. Was it during, before or after the infection? This information should be provided.

Fig. 2 refers to infection, not replication. We agree that infection encompasses multiple steps of the viral cycle. In our experiments, cells were treated with the drugs immediately before viral infection. We have added the information into the Fig. 2 legend.

If the "Golgi" drugs impact egress only (as inferred by the genetic modulation phenotypes), I would expect that at this early time point, the % of infection would not drastically change (as well as intracellular RNA) but that the extracellular infectious titers would decrease. Plaque assays (or TCID50 assays) and RT-qPCR on intracellular viral RNA should be conducted to better understand the impact of drug treatments.

This is a great suggestion! As the reviewer expected, our new BFA time-point assay shows that at early time points, the intracellular RNA levels for S, N and RdRp are not reduced. However, the extracellular N protein (measured by WB) and virial titer (measured by TCID50 assay), which serve as readouts for virion secretion, are significantly decreased (new Fig. 2C-H).

On page 10, it is said that the virus makes three cycles of replication within 24 hours following infection. On what data is this based? This seems a lot. If this is true (and shown in Huh7-ACE2 cells), does the assay of figure 2 measure spread in general? More importantly, despite mentioned, the cell viability data are not provided. It is important to show them to ensure that these concentrations of drugs are not toxic at the tested concentrations.

It has been reported that a single cycle of SARS-CoV-2 infection is approximately 8 hours (Eymieux et al, 2021). Therefore, Fig. 2 represents a multicycle infection, reflecting a composite measure of viral infection and spread. Under the microscope, we did not observe dramatic cell death at the tested concentration. To further assess cytotoxicity, we performed a cell toxicity

assay for the 9 small molecules that inhibit viral infection of Huh7-ACE2 cells. The results show that no or minor cell death was observed with all these compounds (Fig. S3C).

-I appreciated the extensive confocal microscopy analysis performed by the authors, which seems of high quality and overall, very convincing. They clearly show that SARS-CoV-2 infection induces the fragmentation of the Golgi apparatus although it was reported by others before as mentioned by the authors.

We thank the reviewer for the positive comments. We agree that Golgi fragmentation was observed during SARS-CoV-2 infection, as we mentioned. However, our study provides a comprehensive and systematic analysis of the entire host cell endomembrane system in the response to viral infection.

However, it was hard for me to make the functional link between these data and those related to GRASP55 and TGN46 overexpression/knockdown. First, the authors should assess the morphology of the Golgi apparatus in Huh7-ACE2 when GRASP55 is knocked down/out or when TGN46 is overexpressed. Second, in these 2 conditions that favor replication, it should be assessed whether this correlates with Golgi fragmentation. Even if this was probably shown before, it is relevant to show that these genetic modulations induce Golgi reshaping in this particular cell type by confocal microscopy (and ideally electron microscopy).

Thank you for the suggestion. We performed IF analysis to assess Golgi morphology in Huh7-ACE2 cells under conditions of GRASP55 knockdown or TGN46 overexpression. Our results show that GRASP55 depletion disrupts Golgi structure (Fig. S7D), whereas TGN46 expression does not significantly alter the Golgi morphology (Fig. S8D).

-The fact that GRASP55-GFP expression decreases in 293T the cell surface levels of ACE2, the receptor of Spike (Fig S6), raises concern that the effect of GRASP55 is not specific to the virus and suggests that the whole secretory pathway is altered, while an impairment of virus entry should be expected in this cell line. Is there a similar trend in Huh7-ACE2?

Reviewer 1 raised a similar question regarding viral entry efficiency. Fig. S6B, performed in Huh7-ACE2 cells, shows that GRASP55-GFP expression also decreases ACE2 surface level in these cells. To further assess whether GRASP55 expression affects viral entry, we performed RT-qPCR analysis of viral RNA at early time points of infection. We found that authentic SARS-CoV-2 entry efficiency was not altered by GRASP55 expression (new Fig. 5D). Although GRASP55 overexpression does alter the secretory pathway, we want to point out that SARS-CoV-2 infection downregulates endogenous GRASP55 expression. We have used GRASP55 overexpression as a probe to assess the effects of GRASP55 on the secretory pathway and on SARS-CoV-2 virion trafficking, but this does not actually reflect what is observed in SARS-CoV-2 infection.

In addition to addressing the functionality of the secretory machinery in Huh7-ACE2, it would be relevant to repeat the cell surface labelling in the context of pseudotyped virus production with

other viral envelopes such as VSV G protein or HIV gp41/gp120. If the phenotype is specific to Spike trafficking, the cell surface abundance of these alternative viral proteins should not be impacted by GRASP55 overexpression. Otherwise, this would indicate a general effect of on the secretory pathway. Besides, since HIV Gag is directed directly to the plasma membrane during particle assembly without entering the secretory pathway, I am not convinced that upstream alteration on nucleocapsid assembly at the ERGIC should be excluded. Indeed, changes on the S/N ratios are generally mild and I feel that this cannot explain the phenotypes in the extracellular infectious titers.

We have removed the original figure because we acknowledge that HIV Gag is directed directly to the plasma membrane, which is different from the trafficking of SARS-CoV-2 spike protein. We appreciate the reviewer's recognition of the difference in extracellular infectious titers between GFP and G55-GFP expressing cells. We hypothesize that GRASP55 expression not only reduces the number of spikes on each virion but also inhibits the secretion of SARS-CoV-2, resulting in a significantly lower extracellular infectious titer. We agree that it would be interesting to test whether GRASP55 expression affects viral production with other viral envelopes. However, this is beyond the scope of the current study and represents a promising direction for future research.

More generally, the comparison between trafficking and assembly should be better assessed and not simply based on extracellular N and S levels. It was hard to see the differences between the two in terms of phenotypes. The authors should at least measure the intracellular infectivity upon TGN46 and GRASP55 knock/down and overexpression as well as intracellular vRNA abundance as a readout of RNA replication (which is anticipated to remain unchanged).

We thank the reviewer for the valuable suggestions. We performed RT-qPCR analysis of Spike, N, and RdRp at early time points of infection. The new results show that neither GRASP55 expression (new Fig. 5D) nor TGN46 depletion (new Fig. 7R) affects viral RNA abundance at an early infection timepoint (4 hpi). Also, we found that GRASP55 depletion increased intracellular infectivity (new Fig. 6J) while TGN46 depletion did not affect intracellular infectivity (new Fig. 7O), suggesting that GRASP55 modulates viral assembly but TGN46 does not.

-Finally, mechanistic insight about the viral determinants regulating the morphology of the Golgi would significantly strengthen the study.
Fig S6 shows that S expression decreases ACE2 surface levels? If so, could some S mutants be tested? Does it correlate with Golgi fragmentation? Do other viral structural proteins contribute to Golgi morphological alterations?

We thank the reviewer for the suggestions. These are indeed interesting experiments, but we believe that investigating viral determinants of Golgi fragmentation should be pursued by future studies.

In the same line of idea, how GRASP55 and TGN46 regulate replication. The link with Golgi morphology is unclear. Are these proteins hijacked by SARS-COV-2?

Our new data in this revised manuscript more clearly define the stages in the viral infection cycle that are modulated by GRASP55 and TGN46. New Fig. 5D and Fig. 7R show that neither GRASP55 nor TGN46 affects viral entry or early viral replication. However, GRASP55 perturbation modulates viral assembly and secretion, while TGN46 perturbation affects virion secretion but not assembly. Fig. S6C shows that GRASP55 overexpression in the presence of the virus partially rescues Golgi fragmentation. The mechanisms by which GRASP55 and TGN46 are hijacked by SARS-CoV-2 will be explored in the future studies.

Page 13 mentions some relevant mutants that could be assessed in this context and provide mechanistic insights.

It would be interesting to investigate the effects of GRASP55 mutants or specific domains on SARS-CoV-2 trafficking, which we plan to explore in future studies.

Minor comments:
-The signal of calreticulin in Fig. S1 is too low to appreciate it distribution.

We have increased the intensity of calreticulin staining for both uninfected and infected cells in parallel in Fig. S1. Thank you.

-Fig 4K, Q: The differences in LC3 forms levels are not convincing. These results do not allow to draw any conclusion about autophagy, especially considering that this was done at steady-state and that the autophagic flux was not measured. Indeed, a bafilomycin A treatment control would be required to measure the real induction of autophagosomes. Lysosomal degradation inhibition allows the detection of LC3 accumulation.

We agree that additional experiments are needed to demonstrate autophagic flux alteration by SARS-CoV-2. We observed an increase in LC3II/LC3I ratio in infected cells at steady state and did not explore this further, since this is not our main focus of this study. Therefore, we have removed the LC3 blots and quantification from Figs. 4 and S5.

-In the GRASP55 overexpression and TGN46 knockdown studies, associated cell viability should be measured to control that that these genetic manipulations do not induce any cytotoxicity which may impact viral replication.

We appreciate the reviewer’s suggestions. We performed the LDH cytotoxicity assay under SARS-CoV-2 infection with TGN46 depletion or GRASP55 expression. Our new results show that TGN46 depletion or GRASP55 depletion/expression did not induce significant cell death (Figs. 5C, 6L, and 7Q).

-The authors should test the impact of GRASP55 and GRASP65 knock-out on SARS-CoV-2 replication

Investigating the genetic GRASP55 knockout effect on SARS-CoV-2 replication would be valuable. However, ACE2 protein expression in our Huh7-ACE2 cells decreases with cell passages, making knockout construction on this background impractical due to low ACE2 levels and poor viral infection rates. We believe that both our GRASP55 overexpression and depletion assays sufficiently support its role in SARS-CoV-2 trafficking. Future studies will explore GRASP55 knockout in different cell lines.

-The authors should provide more details about the USA-WA1/2020 isolate in the Methods section. Is it related to the "Wuhan" strain or the variant which spread globally in early 2020 (with D614G mutation in Spike).

USA-WA1/2020 was isolated from an oropharyngeal swab from a patient who returned from China and developed COVID-19 on January 19, 2020, in Washington, USA. It is related to the "Wuhan" strain but does not have D614G mutation in spike. Additional details have been added to the Methods section.

-Fig 8: The combined modulation of GRASP55 and TGN46 expressions does not really seem additive to me since a 70% decrease of either protein modulation is observed while the combined condition brings this value to 75% in TCID50 assays. This does not bring much insight to the study in my opinion. I would suggest that the authors consider removing this figure.

We agree with the reviewer’s recommendation and have removed Fig. 8.

Reviewer #2 (Significance (Required)):

General assessment and advance: The study was generally well performed, and the quality of the microscopy and western blot data is good. It was appreciated that all the phenotypes were quantified extensively. However, I have some concerns regarding the interpretations of some of the key conclusions. Moreover, the fact that it was already described by several groups that Golgi is a key machinery for SARS-CoV-2 virion assembly (ERGIC) and secretion dampens my enthusiasm about the study. In addition, the antiviral activity of several tested drugs was also reported elsewhere. A clear mechanism of how SARS-CoV-2 induces a fragmentation of the Golgi would strengthen the study. In the same line of idea, it is unclear how TGN46 and GRASP55 regulate the late steps of the life cycle. The link between SARS-CoV-2-induced Golgi fragmentation and TGN46/GRASP55 is unclear. In my opinion, the data did not allow to clearly discriminate between virion assembly and egress. I was not convinced that it was not simply

due to a general disruption of the secretory pathway (as attested by ACE2 down regulation upon GRASP55 overexpression).

Targeted audience: This study will be of high interest for molecular virologists (not only working on SARS-CoV-2) but could be very well fit into the scope of molecular/cell biology-focused generalist journals

Reviewer expertise: Molecular virology, virus-host interactions (especially involving membranous organelles), SARS-CoV-2, RNA viruses

Reviewer #3 (Evidence, reproducibility and clarity (Required)):

Summary:

Zhang et al. demonstrated in this study that the Golgi apparatus and many other organelles are disturbed by SARS-CoV-2 infection. They focused on the Golgi apparatus and especially on TGN46 and GRASP55 which are both affected differently in their level of expression by the SARS-CoV-2 infection. TGN46 is overexpressed while GRASP55 is decreased in expression. Through different methods overexpression or depletion, the authors nicely demonstrated that modulation of both proteins either increased or decreased particles production. They demonstrated that in absence of GRASP55, SARS-CoV-2 release is increased in the medium. On the contrary, depletion of TGN46 decreases the secretion of SARS-CoV-2 particles.

We thank the reviewer for the accurate summary of our work.

Major comments:

Figure 1: The authors demonstrated that SARS-CoV-2 expression affected the morphology of multiple organelles. Although the results are clear, my concern was that the MOI=1 was really high which indeed would affect the whole cell. To have a less drastic effect on the cell, I would suggest realizing the visualization of some organelles (Golgi, EEA1, Rab7 for example) at a lower MOI=0.1. In addition, it would be nice to verify with a live-dead assay with the MOI=1 if after 24h the cells are still alive, which will confirm that these disturbances are not caused by cells in process of dying.

We thank the reviewer for the excellent suggestions. Investigating how SARS-CoV-2 reshapes subcellular organelles at low MOI (e.g., 0.1) and at different time points would be interesting but is beyond the scope of our study. However, we have performed LDH assay at MOI=1, 2 and 3 for 24 hours to assess cell death. Our results show that LDH release was similar across these conditions (Fig. S5R). We also performed RT-qPCR analysis of Spike, N, and RdRp at early time points of infection. The new results show that neither GRASP55 expression (new Fig. 5D)

nor TGN46 expression (Fig. 7R) affects viral RNA abundance at an early infection timepoint (4 hpi).

Figure 2: The results indicated in that panel are really nice. However, the addition of a virus with drugs could increase the proportion of cell death. For the Figure 2C, I propose that the author use a LDH assay to prove that the decrease in infection is not caused by cell death. In addition, a RT-qPCR would be more appropriate to indicate the infection rate and support the microscopy data.

We thank the reviewer for the positive feedback and suggestions. As recommended, we performed an LDH assay to assess cytotoxicity under 9 small molecules treatment of infected cells. Additionally, we performed RT-qPCR analysis for the BFA time-point treatment assay. No significant cell death was observed under these conditions (new Figs. 2D, and S3C).

Figure 3: The authors should have been consistent and add spike instead of nucleocapsid for GalT. According to the figures, Spike seemed to co-localize more with GM130 than Golgin 245. Data analysis of colocalization between Spike and GM130 should be performed to complete the observation. Are no colocalizations of Spike observed with the other Golgi markers?

We agree with the reviewer that it was ideal if spike and GalT were co-stained. Unfortunately, both our spike antibody and GalT antibody are from rabbit, so co-staining could not be done as GM130/spike. We performed colocalization analysis between Spike and GM130, and the results show that GRASP55 expression did enhance Spike and GM130 colocalization to some extent (new Fig. S6E-F). We only co-stained spike with GM130 and Golgin-245 due to the antibody availability.

Figure 4K: While all the experiments were performed at MOI=1, why is the authors using MOI=2 for the immunoblots. Did they have a different result in protein expression for MOI=1 in HuH cells? if so they should show a blot indicating this result.

We did not perform WB to assess protein expression at MOI=1, but our cell toxicity assay showed that there is no significant difference between MOI=2 and MOI=1.

Figure 5: Viral infection should be indicated using RT-qPCR data analysis to support the microscopy observations.

We performed RT-qPCR analysis (new Figs. 2F, 5D, and 7R) and found that BFA treatment did not reduce viral RNA levels at all three time points. Also, GRASP55 expression and TGN46 depletion did not inhibit viral genome RNA levels within one viral infection cycle. Additionally, our new TCID50 assay results support our microscope observation (new Fig. 7O-P). Thanks for the suggestion.

Review COMMONS

Figure 6: The authors should look at the trafficking of ACE2 and TfR in case of GRASP55 depletion like they did in case of GRASP55 overexpression. It could demonstrate if the virus is using trafficking pathways that are common to the one used by some host receptors to reach the plasma membrane.

Thanks for the excellent suggestion. We performed cell surface biotinylation assay of control and GRASP55-depleted cells. We found that ACE2 and TfR receptor displayed a similar reduction on the cell surface (Fig. S7C), consistent with previous findings that GRASP55 depletion induced Golgi fragmentation and accelerated global conventional protein secretion.

Figure 7: Viral infection assay should also be performed by RT-qPCR. Figure 7H: The immunoblots conditions were performed at MOI=3 this time. The authors should indicate why they did not keep the same MOI conditions. In that case, they should use an intracellular marker for their medium experiment to prove that they isolated proteins that are secreted and not simply released from dead cells. I will also suggest to show LDH assay at MOI=2 and 3 to monitor cell death. Is the Golgi fragmented when GRASP 55 is overexpressed in presence of the virus? Microscopy observations should be performed to reply to this question as it will support their model. The authors suggest that GRASP55 overexpression decreases spike incorporation inside the virion. Can they observe if Spike still colocalizes with GM130 when GRASP55 is overexpressed?

We showed that TGN46 depletion inhibits viral infection by both IF and WB. We further confirmed this through TCID50 assay for both cells and media (new Fig. 7O-P), strengthening our hypothesis.

As we described above, we performed morphological analysis at MOI=1 so that we could observe a significant number of infected cells but minimize cell toxicity. We performed immunoblotting (in Fig. 7H) at MOI=3 to get a good viral infection rate.

As suggested, we also performed LDH assay at MOI=2 and 3 to monitor cell death (new Fig. S2O). Fig. S6C shows that GRASP55 overexpression in the presence of the virus partially rescues Golgi fragmentation. GRASP55 expression did also enhance Spike and GM130 colocalization to some extent (new Fig. S6E-F).

Minor comments:

Figure 1P in the text: Considering that Rab7 up-regulation is equal to "growth of late endosome" is an overstatement. Rab7 is cytosolic at its inactive state and at the endosome at its active state. The authors would have to prove this statement by monitoring an increased quantity of Rab7 at the endosomes which is not enough by just monitoring protein intensity by microscopy. As Rab7 is also localized in lysosomes, and the authors used Lamp2 as a lysosomal marker, it

is strange that the area of these structures is not increased. The authors should replace the term "growth" by "an increase in the area of their vesicles".

We did observe less but larger LAMP2 puncta in the infected cells. We agree with the reviewer and rephrased “growth” by an increase in the area of their vesicles”. Thank you for the excellent suggestions.

Figure 1Q-T: The observations described in the text did not match the quantification, the area of lysosomes is not significantly different from the non-infected conditions.

In Fig. 1Q-T, we did observe fewer but larger LAMP2 puncta in the infected cells, which was consistent with our quantification, i.e., fewer puncta (Fig. 1R), but each punctum was larger (Fig. 1S), and total area was similar.

Figure 8: In the text, it is mentioned that there is "a dramatic reduction of spike and N in the lysate in GRASP55-expressing and TGN46 depleted cells". However, the quantification indicated that the decrease in N and S content is non-significant. Can the authors precise what was the sample of comparison in the text (siControl versus siTGN46 or siTGN46+GFP versus siTGN46+GFP-GRASP55)?

The decrease in N and S content is significant with the lysate sample comparison (siControl versus siTGN46; siControl+GFP versus siTGN46+GFP; siTGN46+GFP versus siTGN46+GFP-GRASP55). We have now removed this Figure following Reviewer #2’s suggestion, since the results are consistent with single protein manipulation and more experiments are needed to confirm whether there is an additive effect.

Referee cross-commenting

I agree with most of the concerns of the other reviewers. I do also consider that they should have done their study on cells expressing naturally ACE2. However, at this stage, it will be a lot of work to perform all of their study in a more relevant cell type. The authors should repeat some of their key experiments in lung-derived cell types, to determine if GRASP55 and TGN46 have the same effect on SARS-CoV-2 virion secretion/production.

We thank the reviewer for the suggestions and understanding. As we mentioned before, our study utilizes Huh7-ACE2 cells, which are sorted for the high expression of endogenous ACE2 protein, without ACE2 overexpression. Actually, we also tested A549 and Calu-3 cells. While A549 cells displayed very low infection rate, Calu-3 cells displayed disorganized Golgi without viral infection. However, we did perform immunofluorescence assays in Calu-3 cells. Consistent with our findings in Huh7-ACE2 cells, SARS-CoV-2 infection disrupts Golgi structure and alters protein levels of TGN46 and GRASP55 in Calu3 cells (new Fig. S5R-W). Also, others have reported that liver can be a target for SARS-CoV-2 infection in humans. Furthermore, we confirmed GRASP55 downregulation and TGN46 upregulation in VeroE6 cells (Fig. S6K-N).

Reviewer #3 (Significance (Required)):

The study identified two Golgi proteins (TGN46 and GRASP55) that are involved in modulating the release of SARS-CoV-2 particles from the cells. As these proteins are also acting on general secretion of host proteins to the plasma membrane, the effect on SARS-CoV-2 release could just be indirect. However, it does not change the informative points of the study raised by Zhang et al. It highlights really well how the host trafficking pathway could be diverted for the purpose of the virus, which is to produce particles to maintain its survival.

Strengths: The authors performed a precise and well quantified study. Observing how SARS-CoV-2 impacts host organelles morphology and uses host trafficking proteins to produce particles, brings more clarity on some unclear parts of the life cycle of the virus. In addition, it exposes new targets for therapeutic studies.

We thank the reviewer for the positive comments.

Weakness: The paper is mostly based on microscopy analysis and need some other methods to support their data. The paper lacks some molecular mechanisms explaining the clear role of GRASP55 and TGN46 in particle production or assembly.

In the revised version, we incorporated RT-qPCR assay, cell cytotoxicity assay, and BFA time-point treatment assay. Notably, we added intracellular and extracellular viral titer assays to more precisely distinguish between effects on virion assembly and virion secretion. We also confirmed the key observation that SARS-CoV-2 infection modulates GRASP55 and TGN46 expression in the Calu-3 lung cell line. Additionally, our early time-point results clearly support the role of GRASP55 and TGN46 in viral trafficking.

- Audience: The paper will be interesting for basic research for a virology and cell biology audience.
- Field of expertise with a few keywords: Virology and host cell trafficking.

References

Barnes E (2022) Infection of liver hepatocytes with SARS-CoV-2. *Nat Metab* 4: 301-302

Bekier ME, 2nd, Wang L, Li J, Huang H, Tang D, Zhang X, Wang Y (2017) Knockout of the Golgi stacking proteins GRASP55 and GRASP65 impairs Golgi structure and function. *Mol Biol Cell* 28: 2833-2842

Eymieux S, Rouille Y, Terrier O, Seron K, Blanchard E, Rosa-Calatrava M, Dubuisson J, Belouzard S, Roingeard P (2021) Ultrastructural modifications induced by SARS-CoV-2 in Vero cells: a kinetic analysis of viral factory formation, viral particle morphogenesis and virion release. *Cell Mol Life Sci* 78: 3565-3576

Ghosh S, Dellibovi-Ragheb TA, Kerviel A, Pak E, Qiu Q, Fisher M, Takvorian PM, Bleck C, Hsu VW, Fehr AR *et al* (2020) beta-Coronaviruses Use Lysosomes for Egress Instead of the Biosynthetic Secretory Pathway. *Cell* 183: 1520-1535 e1514

Hoffmann M, Hofmann-Winkler H, Smith JC, Kruger N, Arora P, Sorensen LK, Sogaard OS, Hasselstrom JB, Winkler M, Hempel T *et al* (2021) Camostat mesylate inhibits SARS-CoV-2 activation by TMPRSS2-related proteases and its metabolite GBPA exerts antiviral activity. *EBioMedicine* 65: 103255

Hoffmann M, Mosbauer K, Hofmann-Winkler H, Kaul A, Kleine-Weber H, Kruger N, Gassen NC, Muller MA, Drosten C, Pohlmann S (2020) Chloroquine does not inhibit infection of human lung cells with SARS-CoV-2. *Nature* 585: 588-590

Xiang Y, Wang Y (2010) GRASP55 and GRASP65 play complementary and essential roles in Golgi cisternal stacking. *J Cell Biol* 188: 237-251
