# Supplementary material for: SARS-CoV-2 remodels the Golgi apparatus to facilitate viral assembly and secretion
Source: PLoS Pathog. 2025 Jun 20;21(6):e1013295. doi: 10.1371/journal.ppat.1013295 (PMC12208438; doi:10.1371/journal.ppat.1013295)
Supplement: Attachment — Submitted filename: Response to Reviewers.pdf [file ppat.1013295.s015.pdf]

Reviewers' Comments:

Authors' Responses to Reviewers' Comments

Part I - Summary

Please use this section to discuss strengths/weaknesses of study, novelty/significance, general execution and scholarship.

Reviewer #3: Thank you for responses to my review. While the abundant data, the careful experimentation, and the important topic are all very impressive, I remain skeptical of some data interpretations. Note, for example, the reviewer 3 response #5 that addresses effects of BFA on viral protein levels. The response includes a very clean WB showing that BFA dramatically reduces intracellular N proteins. Therefore it is not surprising that there is less secreted virus in response to BFA. Two points here; first, the WB that is presented in responses to reviewers should be in the paper.

We thank Reviewer #3 for the positive comments and helpful suggestions.
In response to the reviewer's recommendation, we have added the western blot images and corresponding quantification results (Fig. S3G-K) to the revised manuscript.

Second, perhaps more to the point; the overall findings point to BFA blocking N translation or stability, making it hard to interpret findings in context of virus assembly. The authors claim that BFA is blocking virus "assembly" but the data appear to indicate that the BFA effect is at some earlier infection stage.

Based on our results, BFA treatment did not inhibit viral genomic RNA replication (Fig. 2F), but significantly decreased viral titer both intracellularly and extracellularly (Fig. 2G-H). N protein levels were reduced in both cell lysates and culture media (Fig. 2E; Fig. S3G-J). Notably, both intracellular Spike and N proteins are reduced upon BFA treatment, with N protein showing a more pronounced reduction than spike (Fig. S3G-J).

We agree with the reviewer that BFA likely also affects global protein synthesis, including viral protein synthesis, in addition to inhibiting virus assembly and release. It has been reported that BFA blocks protein secretion and inhibits protein synthesis, although the inhibitory effect is generally stronger on secretion than on translation (Ripley *et al*, 1993). Consistent with this,

after 6 h of BFA treatment, N was not detectable in the medium but remained visible in the cell lysate (Fig. S3G & Fig. 2E, lane 4), suggesting that BFA disrupts N protein secretion more than its synthesis and/or stability.

To better address the reviewer's concern, we quantified the medium-to-lysate ratio of N protein over the time of BFA treatment as a proxy for secretion efficiency. As shown in Fig. S3K, this ratio is significantly reduced after 6 h BFA treatment, supporting the idea that BFA disrupts the trafficking and release of viral proteins.

We thank Reviewer #3 for the insightful comment. In response, we have revised the text and included new data and a relevant reference to clarify the interpretation.

The above findings related to BFA and effects on N proteins relates to this reviewer's separate suggestion to plot data in terms of media to lysate (M/L) ratios. If this were done for N proteins, i.e., in Fig 2 (and with the additional WB that was provided in response to reviewers), then the M/L ratios would suggest that BFA is not changing M/L ratios and therefore is not directly affecting virus secretion. Hence I stick with the suggestion that M/L ratios have value in data interpretation. The authors note correctly that both unassembled and virus-assembled proteins are present in lysates, while media contain mostly virus-assembled proteins. However, this is not really a confounder in analysis. If GRASP55 and TGN46 are directly affecting virus assembly and secretion, and not other infection stages, then they would be expected to change viral protein M/L ratios. I think the M/L ratios have value in data interpretation.

We thank Reviewer #3 for the thoughtful suggestion regarding M/L ratio quantification in the BFA assay. In response, we have quantified the M/L ratio of N protein, and our new data show that this ratio is significantly reduced after 6 h BFA treatment. This result is consistent with our western blot analysis, which shows that N protein is undetectable in the medium but remains detectable in the cell lysate under these conditions. Due to the absence of detectable spike protein in the medium, the M/L ratio for spike could not be reliably determined in this assay. We agree with the reviewer that M/L ratios offer valuable insight into viral protein trafficking and secretion, and we hope that our new results and quantification adequately address this point.

Part II – Major Issues: Key Experiments Required for Acceptance

Please use this section to detail the key new experiments or modifications of existing experiments that should be absolutely required to validate study conclusions.

Generally, there should be no more than 3 such required experiments or major modifications for a "Major Revision" recommendation. If more than 3 experiments are necessary to validate the study conclusions, then you are encouraged to recommend "Reject".

Reviewer #3: (No Response)

Reference

Ripley CR, Fant J, Bienkowski RS (1993) Brefeldin A inhibits degradation as well as production and secretion of collagen in human lung fibroblasts. J Biol Chem 268: 3677-3682
