# Supplementary material for: SARS-CoV-2 remodels the Golgi apparatus to facilitate viral assembly and secretion
Source: PLoS Pathog. 2025 Jun 20;21(6):e1013295. doi: 10.1371/journal.ppat.1013295 (PMC12208438; doi:10.1371/journal.ppat.1013295)
Supplement: Attachment — Submitted filename: Response to Reviewers.docx [file ppat.1013295.s014.docx]

**Reviewers' Comments:**

**Authors’ Responses to Reviewers' Comments**

Part I - Summary

Please use this section to discuss strengths/weaknesses of study, novelty/significance, general execution and scholarship.

Reviewer #1: The authors have addressed all my comments. I am positively impressed by the quality of the revised manuscript. I do not have any additional concern.

We thank Reviewer #1 for the positive comments on our revised manuscript.

Reviewer #2: Zhang et al. demonstrated in this study that the Golgi apparatus and many other organelles are disturbed by SARS-CoV-2 infection. They focused on the Golgi apparatus and especially on TGN46 and GRASP55 which are both affected differently in their level of expression by the SARS-CoV-2 infection. TGN46 is overexpressed while GRASP55 is decreased in expression.

The authors nicely demonstrated that modulation of both proteins either increased or decreased particles production. They demonstrated that particle secretion was impacted in absence of TGN46 suggesting that SARS-CoV2 divert specific host pathways for its release at the plasma membrane. GRASP55 depletion at the contrary, increases viral secretion, although it is still unclear how the fragmentation of the Golgi apparatus could play a role in increasing particle secretion. The study is interesting and highlight the involvement of the Golgi apparatus in SARS-CoV2 life cycle with key players identified.

We thank Reviewer #2 for the positive and precise summary of our manuscript.

Reviewer #3: SARS-CoV-2 remodels the Golgi apparatus to facilitate viral assembly and secretion

This submission documents Golgi fragmentation in SARS-CoV-2 infected cells, thereby confirming previous reports. The study proceeds by treating infected cells with various chemicals that are known to perturb protein synthesis and trafficking, with focus on BFA, a drug that collapses Golgi and has been tested for inhibition of coronavirus MHV secretion. Unlike MHV, BFA blocked SARS-CoV-2 infection. There are then EM images that are used to support statements that SARS-CoV-2 particles reside in Golgi vesicles. The works then advance to GRASP55 and TGN46 because these two Golgi proteins were discovered as reduced and elevated, respectively, in IFA images of infected cells. GRASP55 was then forcibly increased or decreased by transgene expression or siRNA KD. TGN46 was decreased by siRNA KD. Increased GRASP55 did modestly reduce SARS-CoV-2 infection while decreased GRASP55 increased infection. Decreased TGN46 modestly decreased SARS-CoV-2 infection. From these findings, the authors primary conclusion is that the Golgi is required for SARS-CoV-2 secretion.

The results are interesting and potentially valuable because they address current questions about the morphogenesis and egress of coronaviruses particles from infected cells. This study adds some insights into the possible roles for Golgi vesicles in virus secretion. While the authors have responded to three “review commons” recommendations, there are still several concerns with some data interpretations and conclusions. There are also some suggestions for manuscript modification.

We thank Reviewer #3 for the overall positive comments and valuable suggestions that helped improve the interpretation of our results.

Part II – Major Issues: Key Experiments Required for Acceptance

Please use this section to detail the key new experiments or modifications of existing experiments that should be absolutely required to validate study conclusions.

Generally, there should be no more than 3 such required experiments or major modifications for a "Major Revision" recommendation. If more than 3 experiments are necessary to validate the study conclusions, then you are encouraged to recommend "Reject".

Reviewer #1: (No Response)

Reviewer #2: The study was nicely improved from the first version of the manuscript. Most of the experiments suggested, were performed and carefully done. The authors supported their microscopy observations with RT-qPCR, confirmed that their observations were not caused by cell death using LDH assays. The biochemical studies to observe particles secretion and the disturbance caused by the virus in host cell receptor localization at the plasma membrane was convincing.

We thank Reviewer #2 for recognizing the improvements in our revised manuscript.

Reviewer #3: Comments:

1. Much of the report seems to be framed around a prior publication in which secretion of the coronavirus MHV was reported to bypass the conventional Golgi secretory pathway (Cell 183: 1520-1535 e1514). Given this perspective, it would seem that SARS-CoV-2 and MHV should be compared directly. Huh-7 – MHV receptor-positive cells can be infected by MHV.

We agree with Reviewer #3 that it would be interesting to test whether BFA treatment inhibits the assembly and trafficking of other coronaviruses like MHV. Our study focuses specifically on the assembly and secretion of SARS-CoV-2. The secretion pathway of other types of coronaviruses will be explored in future studies. We have now added the sentence “It would be interesting to investigate whether BFA treatment similarly inhibits the assembly and trafficking of other coronaviruses.” in the Discussion of the revised text.

2. While data do show that GRASP55 increases/decreases will decrease/increase SARS-CoV-2, the effects appear modest. Similarly, decreased TGN46 appears to modestly decrease SARS-CoV-2 infection and in the TGN46 case, this reviewer sees little evidence that the effect is at the level of virus secretion. The data in Figs 5, 6 and 7 should be plotted differently, specifically focusing on viral proteins and infectious virions in media vs. lysates. Plotting media/lysate ratios in the case of TGN46 KD might show that TGN46 is reducing infection at stages that are not related to assembled virion secretion (in particular, Fig 7F and 7OP data are not convincingly showing that TGN46 KD impairs virus secretion). Plotting media/lysate ratios for S and N proteins may more convincingly help to determine whether GRASP55 operates at the level of spike protein incorporation into virus particles. Furthermore, M proteins (not N proteins) are the principal coronavirus proteins that operate in virus assembly and the S and M protein ratios may be more illuminating than S and N ratios.

Figure 6 J-K and Figure 7 O-P show that within one virion cycle, virus secretion was significantly altered by manipulating the protein levels of GRASP55 or TGN46. Although our knockdown efficiency appears to be adequate, complete knockout of GRASP55 or TGN46 is expected to show even greater effects. Therefore, we believe it is convincing to conclude that manipulation of GRASP55 or TGN46 protein levels affects virus secretion.

For the western blots in Figs 5, 6, and 7, viruses from the culture media were enriched by PEG virus precipitation, while viruses from cell lysates were not enriched. S and N proteins detected in the media represent assembled virion particles, whereas those from cell lysates include both assembled virion particles and unassembled viral proteins. Therefore, we do not agree that comparing media to lysate ratios is a better approach. Additionally, the N protein (the only cytosolic structural protein) binds to viral RNA, and we consider it a suitable control for assessing S incorporation. Since S, M, and E are all transmembrane proteins, it would be interesting to examine whether M and E incorporation are also similarly affected by GRASP55 protein level manipulation. These remain open and intriguing questions deserving of future exploration.

3. The word “expression” is used throughout the paper in reference to changes in GRASP55 and TGN46 protein levels in virus-infected cells. This gives the impression that virus infections are altering GRASP55 and TGN46 gene expressions to facilitate virus assembly and secretion, but gene expressions were not tested after virus infection. Might the altered GRASP55 and TGN46 levels be due to altered protein stabilities following infection, perhaps due to prior Golgi fragmentations? If this remains a possibility, it should be noted and text changed accordingly.

We thank Reviewer #3’s suggestion that “expression” is not the best option. To clarify, we added the sentence in the Discussion section “To be noted, the mechanisms underlying SARS-CoV-2-induced GRASP55 downregulation and TGN46 upregulation - whether through altered gene expression or protein stability - remain unclear and warrant further investigation.”

Minor comments:

1. Figs 1E-H; is GPP130 or GRASP65 being imaged? (legend says GRASP65)

We thank the Reviewer for pointing out this oversight. The figure is correct, and we have revised the legend accordingly.

2. Figs 2AB; infection “rates” not measured, rather, percent infected cells are measured – consider rephrasing text and ordinat axes

We thank the Reviewer for the suggestion. We have revised it to “percentage.”

3. Figs 2AB; should note that many of the chemical conditions lack controls (cannot be certain whether some chemicals are active under any condition). Consider removing the data on those drugs that were found to have no effect on infection and were also not shown to have any effects on events in which they should be effecting changes.

We believe that those chemicals without inhibitory effects serve as additional controls and we have used the same concentration as in many other assays that we know they work.

4. Fig 2D; should have a positive control condition for LDH release (this applies to all of the data using LDH release as a measure of compromised cells)

For all LDH release assays, medium collected from control cells treated with 0.2% Triton X-100 for 15 minutes was used as a positive control and set as 100%.

5. Fig 2E; should show intracellular N in comparison to the N in media

The intracellular N blot shows that N protein levels are also decreased upon BFA treatment (see figure below). Additionally, Fig. 2F indicates that the mRNA level for N is not reduced, suggesting that viral genome replication is not inhibited by BFA treatment. These results suggest that inhibition of viral assembly lowers the intracellular level of N proteins but not viral genome replication, which warrant future investigation.

6. Fig 2C-G; should include MHV-infected cells as comparative control, especially given the importance of this finding and given that the text highlights potential distinctions between SARS-CoV-2 and the previous findings reporting MHV resistance to BFA.

We agree with Reviewer #3 that it would be interesting to investigate whether BFA treatment inhibits the assembly and trafficking of SARS-CoV-2 and other coronaviruses like MHV. While the main focus of our study is on SARS-CoV-2 assembly and secretion, the secretion pathways of other coronaviruses remain interesting questions for future work. We have now added the sentence “It would be interesting to investigate whether BFA treatment similarly inhibits the assembly and trafficking of other coronaviruses.” in the revised Discussion.

7. Fig 3A-E; is GRASP65 or GM130 being imaged? (legend says GM130)

We thank the Reviewer for pointing out this oversight. The figure is correct, and we have corrected the legend.

8. Fig 3P; can agree that white arrowheads are probably pointing to viral particles, but how can it be claimed that black arrows and arrowheads are pointing to golgi membranes and DMVs, respectively?

The Golgi membranes, indicated by black arrows, exhibit a pattern similar to the fragmented Golgi in the third row of Supplemental Fig. 4. The DMVs, marked by arrowheads, displayed thicker membranes (almost twice the thickness of other organelle membranes) and were observed only in infected cells.

9. Fig 4; “down and up-regulate” may not be best terms for results that show host protein levels within infected cells by WB (see major comments)

Thanks for the suggestion. We have added the explanation “To be noted, the mechanisms underlying SARS-CoV-2-induced GRASP55 downregulation and TGN46 upregulation - whether through altered gene expression or protein stability - remain unclear and warrant further investigation.” it in the revised text.

10. Fig 6; the word “accelerates” in the Fig 6 titles is not appropriate, given that kinetic analyses of virus assembly and secretion were not performed.

Thanks for the suggestion. We have revised it to “promotes” in the text.

11. Fig 7; the word “required” in the Fig 7 title is not appropriate, given the modest effects of TGN46 KD on SARS-CoV-2 infection.

Thanks for the suggestion. We have changed the sentence in the text.

12. Discussion, line 460; “integrity of conventional trafficking pathway is essential for SARS-CoV-2..” This statement is puzzling because the data show that the conventional trafficking pathway is disturbed by Golgi fragmentation.

Thanks for the suggestion. We have deleted “integrity of” from the text.
